# Psychological mechanism of acceptance and commitment therapy and rational emotive behavior therapy for treating hoarding: Evidence from randomized controlled trials

**DOI:** 10.3389/fpubh.2023.1084467

**Published:** 2023-02-10

**Authors:** Shuanghu Fang, Dongyan Ding, Ru Zhang, Mingjie Huang

**Affiliations:** School of Educational Science, Anhui Normal University, Wuhu, China

**Keywords:** acceptance and commitment therapy, rational emotive behavior therapy, hoarding, psychological flexibility, mediation

## Abstract

Hoarding is a common problem behavior worldwide and is detrimental to the physical and mental health of individuals and groups. Currently, effective interventions for hoarding are cognitive-behavioral therapies, but their post-intervention efficacy is questionable, and the available research does not examine the mediating variables of the effects of interventions on clinical outcomes. Moreover, current research on hoarding has focused on Western countries. Therefore, there is a need to investigate the efficacy of other forms of cognitive behavioral therapy on hoarding as well as other psychological outcomes related to hoarding and mediating variables that contribute to its effectiveness in different cultural contexts. One hundred thirty-nine college students with higher hoarding behaviors were randomly divided into three groups: 45 in the Acceptance and Commitment Therapy (ACT) group, 47 in the Rational Emotive Behavior Therapy (REBT) group, and 47 in the control group. They completed the Saving Inventory-Revised (SI-R), Obsessive-Compulsive Symptom Scale (OCSS), Difficulties in Emotion Regulation Scale (DERS), Experiences in Close Relationships Inventory-Attachment Anxiety Subscale (ECR), Depression Anxiety Stress Scales (DASS-21), Acceptance and Action Questionnaire II (AAQ-II), and Cognitive Fusion Questionnaire (CFQ) before and immediately after the intervention. The results showed that ACT and REBT improved individuals' psychological flexibility, cognitive fusion, acquisition-difficulty discarding, clutter, negative affect (anxiety, depression, stress), attachment anxiety, obsessive-compulsive disorder, and difficulty in emotion regulation compared to the control group. In addition, ACT was more effective than REBT in improving psychological flexibility and reducing hoarding, cognitive fusion, depression, stress, and obsessive-compulsive disorder; there were no significant differences between the two in anxiety and emotion regulation difficulties. Furthermore, psychological flexibility is a mediator of the effect of ACT and REBT on some behavioral and psychological outcomes (hoarding, negative affect, attachment anxiety). Limitations were discussed.

## Introduction

Hoarding disorder has been considered a subtype of obsessive-compulsive disorder (OCD) and obsessive-compulsive personality disorder (OCPD) (1). Hoarding is characterized by severe difficulties in discarding possessions, resulting in a cluttered living space that cannot be used for its intended purpose (2, 3). Frost and Hartl (3) identified hoarding as consisting of the following key elements: the acquisition of a large number of possessions, the subsequent failure to discard the possessions, and the clutter that results in a living space that cannot be used in the manner for which it was designed. Studies have shown that up to 5.8% of the population has a hoarding disorder (4). Due to cultural differences, in China, hoarding may be considered a good virtue of “frugality” and “thriftiness”. As a result, hoarding is common in China. Previous research has shown that ~51.8% of college students reported hoarding behaviors, but only about 4.22% met the DSM-5 diagnostic criteria for hoarding disorder (5). Severe hoarding behaviors can even lead to community health and safety problems, such as falls, fires, and death (6). Additionally, hoarding is an important mental health problem that can lead to severe personal distress and may result in impairment of social skills, occupational abilities, and family functions (7, 8). Individuals with higher hoarding behaviors have poorer information processing skills (9, 10) and may also have problems with attention and memory (11–13).

Research has shown that individuals with higher attachment anxiety often exhibit higher hoarding (14, 15), which is consistent with insecure attachment (16). Attachment theory suggests that the severe difficulty of discarding possessions in hoarding may be an attempt to compensate for loneliness and lack of emotional connection with people (17, 18). Emotional over-attachment to objects appears to be one of the distinguishing features of hoarding behaviors, with possessions often seen as an extension of the self (19). Attachment anxiety is a significant predictor of hoarding behavior (20, 21). It has been suggested that intolerance of uncertainty and experiential avoidance exerted sequential mediating effects on the association of attachment anxiety with hoarding behavior, i.e., attachment anxiety could influence hoarding behavior, which in turn affected intolerance of uncertainty and experiential avoidance and then impacted hoarding behavior (22). Therefore, examining the mechanism of attachment anxiety on hoarding behavior from the perspective of cognitive and coping means has implications for the prevention and intervention of hoarding behavior.

Many theoretical models have integrated emotional regulation and emotional responses into the onset and maintenance of psychological symptoms (23, 24). Individuals with hoarding experience difficulties with emotional regulation (13), and they also have higher levels of experiential avoidance and emotion regulation difficulties than healthy individuals (25). Systematic reviews and meta-analyses suggested that emotion dysregulation had a medium to strong association with hoarding (26, 27). Hoarding disorder is considered a subtype of OCD, and the cognitive model of OCD suggests that OCD is influenced by emotion regulation difficulties and consistently implicates the nonacceptance of emotions and difficulties engaging in goal-directed behavior when distressed (28). Some researchers have suggested that emotion regulation can play an important role in understanding OCD and in improving treatment, as well as improving the tolerability of treatment for individuals with this debilitating disorder (28). Emotional reactions also play a key role in the onset and maintenance of hoarding (26). Hoarding is closely related to the experience of negative emotions (29, 30). Previous research reported that people with higher levels of hoarding may also suffer from negative affect (e.g., depression, anxiety, stress) (13, 30). The cognitive-behavioral model of hoarding highlights the role of strong negative emotional responses (e.g., sadness, anxiety, depression) in stimulating hoarding behaviors (3, 24). Some research has argued that stronger negative emotions after viewing emotional movies were associated with more severe hoarding symptoms (24, 31). Some studies have suggested that it is appropriate to examine whether emotional responses should be the target of behavioral interventions for hoarding (24).

Hoarding could be influenced by psychological flexibility. As a core component of acceptance and commitment therapy (ACT), psychological flexibility can be divided into six core processes: acceptance, cognitive defusion, engagement with the present moment, self as context, values, and committed action (32–34). Psychological flexibility can assist individuals in consciously accepting adverse life events and adversities with an open mindset and help people persist and act on their value-consistent goals (35, 36). Existing meta-analyses suggest that psychological flexibility is positively related to individuals' mental health and adaptive behaviors and negatively related to individuals' negative emotional affect and problematic behaviors (37–39). Psychological flexibility has been found to be significantly associated with hoarding behaviors (40). It was found that the lower the degree of psychological flexibility, the higher the hoarding behaviors (31). There was also a significant negative association between psychological flexibility and difficulty discarding and over-acquisition in a clinical sample (41). Some researchers believed that the symptoms of hoarding (e.g., acquisition and clutter) stem from a variety of avoidance behaviors designed to avoid the pain of making poor decisions about possessions (3). Researchers have observed a pattern of behavioral avoidance in most hoarders (3, 25). Experienced avoidance is a subcomponent of psychological inflexibility (33). Psychological inflexibility may manifest as a confusion of thoughts with reality, leading to hoarding (42). Cognitive fusion, as one factor of psychological inflexibility, may further contribute to hoarding (43). If individuals are filled with thoughts about their possessions, such as “I cannot get rid of this,” they may build a narrow repertoire of options for saving or acquiring. The role of cognitive fusion may be related to the emergence of hoarding-related thoughts (43).

Most current interventions for hoarding behaviors are less effective or even ineffective, but many kinds of cognitive behavioral therapy (CBT) have shown definite effectiveness in reducing hoarding disorders (44, 45). However, a meta-analysis indicated that in most cases, patients continue to have significant hoarding symptoms after treatment with CBT (46). CBT has gone through several different eras, generations, or waves. Traditional CBT focuses on modifying maladaptive thought and cognitive distortion patterns in emotions and behaviors, training adaptive thinking, and engaging in enjoyable activities (47, 48). Third-wave CBT is based on the concept of context and focuses more on the person's relationship with thoughts and emotions than on the content of cognitions (47). Therefore, it is necessary to explore the role of other forms of CBT that are more effective for treating hoarding behaviors and related outcomes.

Rational Emotive Behavior Therapy (REBT) is one kind of traditional CBT, but there is little empirical evidence on the efficacy of REBT for hoarding. The theoretical basis of REBT is the ABC theory of emotions (49, 50). In the ABC model, A represents the activating event, B represents rational or irrational beliefs, and C represents emotional, cognitive, and behavioral consequences (49). According to the ABC model, when the reality of a situation contradicts our needs, we transform our rational needs into inflexible desires, and therefore we become emotionally upset (51). One of the core principles of REBT is that these evaluative beliefs influence people's perceptions of events and then affect their emotions, behaviors, and reactions to those events (52). The primary goal of REBT is to change irrational beliefs through cognitive restructuring and to foster rational beliefs to promote mental health and wellbeing (49, 52). A systematic review and meta-analysis summarized the effectiveness and efficacy of 50 years of REBT and concluded that REBT has a medium effect size compared to other interventions on irrational beliefs and different types of outcomes (e.g., emotional outcomes, behavioral outcomes, cognition outcomes, and health outcomes) (53). Currently, REBT has been used with good results in the treatment of psychological disorders, such as depression (54) and social phobia (55). However, no studies have explored the utility of REBT for hoarding. Hoarding is a subtype of OCD (1). A case study of schizophrenia and OCD in China concluded that REBT has an ameliorative effect on OCD (56). REBT specializes in helping clients unconditionally self-accept their OCD suffering, as well as increasing their frustration tolerance about affiliation (57). Therefore, we hypothesized that REBT may be effective in alleviating hoarding behaviors.

According to the cognitive model of hoarding, the manifestation of hoarding (e.g., difficulty discarding, clutter, and acquiring) stems from a variety of avoidance behaviors aiming to avoid distress (3). Acceptance commitment therapy, as a third-wave CBT, can reduce experiential avoidance by increasing psychological flexibility while encouraging participants to make behavioral changes consistent with their values (35, 58). Hoarding behaviors are considered lifelong, and therefore, treatment should focus on symptom improvement rather than complete remission (46, 59), which is consistent with the notion of not changing symptoms but only the relationship with distress in the ACT. Some meta-analyses and reviews have found that ACT has efficacy for improving mental health and reducing behavioral disorders in adults and children (60–62), such as decreasing anxiety, depression, stress, eating disorders (63), obsessive-compulsive disorder (64), and smoking (65). A recent intervention trial with six individuals revealed the effectiveness of ACT for reducing hoarding symptoms (66), but the participants in this trial were all white women. One of the authors of this study explored the efficacy of an ACT self-help program on hoarding (67) and concluded that ACT has an ameliorative effect on hoarding. However, to date, the efficacy of ACT on hoarding in China has not yet been explored.

Currently, the vast majority of ACT research or psychological flexibility studies have been conducted in Western countries and in English. In addition, contemporary research on hoarding has also focused on Western countries, and there is a lack of research on the effects of ACT on hoarding behaviors in different cultural contexts. Moreover, some studies focusing on mechanisms of change have suggested that the effect of ACT or CBT on outcome variables (e.g., depression, pain interference, and OCD) is mediated by its impact on psychological flexibility or cognitive fusion (68–70). However, previous studies on the effects of CBT on hoarding have failed to examine the possible mediating role of psychological flexibility or cognitive fusion as a process variable. A test of mediating effects would be helpful in understanding the mechanisms of CBT in non-Western countries and the role of psychological flexibility and cognitive fusion in individual psychological and behavioral changes.

According to the aforementioned, hoarding can be accompanied by attachment anxiety, difficulties with emotion regulation, and negative emotions. Therefore, the main purpose of this study was to investigate the efficacy of ACT and REBT on hoarding among Chinese college students and to explore whether psychological flexibility or cognitive fusion mediates the effect of ACT/REBT on the outcome variables (i.e., hoarding behaviors, difficulties in emotion regulation, and attachment anxiety).

## Methods

### Participants

**Figure 1:**
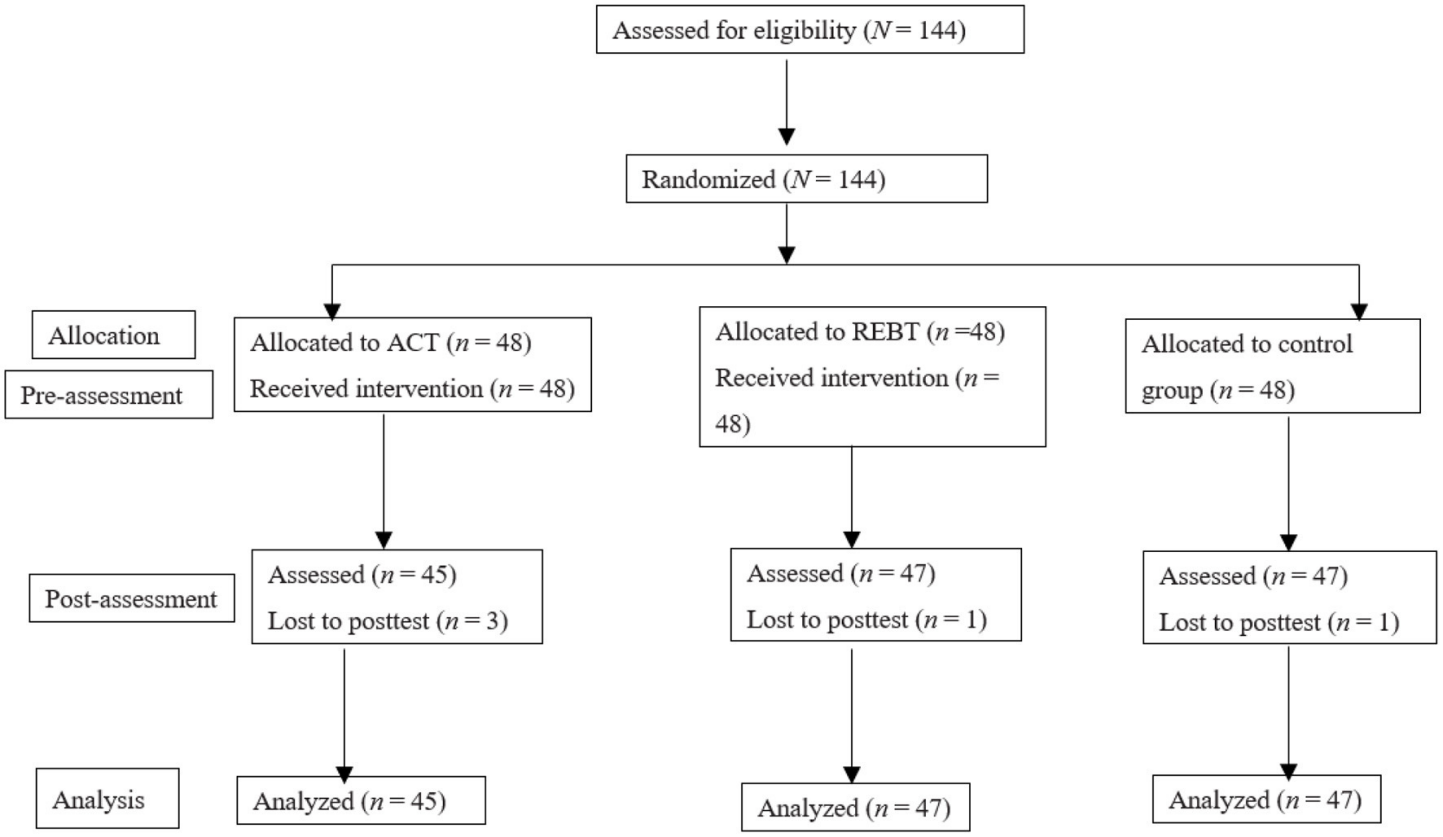
Flowchart of participants.

This study was approved by the Ethical Committee of Anhui Normal University. A total of 1,620 questionnaires were distributed to university students, and 14,78 valid questionnaires were returned (91.2% effective rate). Among the 1,478 surveys, 533 college students had high hoarding problems (total score of Saving Inventory-Revised above the cutoff of 42). Participants in this study were recruited from these 533 university students. The inclusion criteria of the intervention group were being aged 18 years or older and having a self-reported total score on the Saving Inventory-Revised above the cutoff of 42. Exclusion criteria assessed *via* self-report measures and one-on-one online semistructured interviews with a trained research assistant were suicidal intentions, use of psychiatric medicines, receiving psychological counseling or other treatment, and not being willing to undergo the intervention. In this way, we recruited 144 participants. They were randomly divided into three groups: the ACT group, REBT group, and control group, with 48 participants in each group (see Figure 1). It is necessary to note that these students did not participate in other interventions or take medication to alleviate their symptoms. Informed consent was obtained from all students. Five participants withdrew from the intervention for personal reasons (i.e., no time to complete all intervention programs or questionnaires) in the process, resulting in a total sample of *N* = 139, of which 66 (48.2%) were male and 73 (51.8%) were female, with a mean age of 19.65 ± 1.46 years. There were 45 participants in the ACT group, including 22 males and 23 females, with a mean age of 19.71 ± 1.56 years; 47 participants in the REBT group, including 23 males and 24 females, with a mean age of 19.72 ± 1.49 years; and 47 participants in the control group, including 21 males and 26 females, with a mean age of 19.53 ± 1.37 years.

To test the reasonableness of the sample size, statistical power was calculated using G^*^Power 3.1 software (71). Referring to the parameter settings in previous studies (72), the covariance analysis was chosen with an effect size of 0.4, a significance level of 0.05, a sample size of 139, a group size of 3, and a covariate of 1. The statistical power was 0.99, which exceeded the basic level of 0.80, so the sample size of this study met the requirements.

### Procedure

This study was a three-group randomized controlled trial. Participants were randomly assigned to one of the three groups. The ACT group was given acceptance commitment therapy group counseling, the REBT group was given rational emotional behavior therapy group counseling, and the control group was left untreated. The ACT, REBT, and control groups were pre-tested before the beginning of group counseling and post-tested after the completion of group counseling.

### Interventions

Group interventions for both groups were conducted once a week for 1.5 h for 8 weeks. The group intervention protocols were designed by two professionally trained psychology graduate students supervised by the first author of this paper, who is the President of the China chapter of Association for Contextual Behavioral Science and is a professionally trained and certified counselor, trainer, and supervisor. The intervention protocols were revised and refined based on the suggestions of the relevant counselors and supervisors. Before each intervention, the first author explained and illustrated the intervention protocol and intervention points and led the interventionists to discuss the intervention process and intervention methods together. After each intervention, the interventionists reported to the first author of this paper, who provided supervision. The entire process of the group intervention was completed under the supervision of the first author.

#### ACT condition

The intervention content for the ACT group was based on the psychological flexibility model, with reference to the book *Acceptance and commitment therapy: The process and practice of mindful change* (73), and *Act in practice: Case Conceptualization in Acceptance and Commitment Therapy* (74). 8-week intervention programs were designed. See Table 1 for the specific intervention programs.

**Table 1:** Acceptance and commitment therapy intervention program.

**Theme**	**Purpose of the activity**	**Main activities**	**Homework**
1. You and I get together	1. Explain the purpose, format and principles of group activities and familiarize members with each other 2. To gain an understanding of acceptance and commitment therapy	1. To familiarize members with each other through activities such as “group hugging” and “snowballing” 2. Clarify the purpose of group activities 3. Introduction to ACT matrix	Read about acceptance commitment therapy.
2. Experience the present moment	Understand what mindfulness is and what positive effects it has on people	1. Warm-up exercise 2. Preliminary study of the basic theory of positive thinking and experiential positive thinking training 3. Positive thinking breathing and body scanning 4. Eating raisins with mindfulness	Breathe with mindfulness and try walking with mindfulness, eating with mindfulness, etc.
3. Acceptance of oneself	1. Feel the painful feelings generated by avoidance and rejection 2. Promote self-acceptance and allow oneself to have painful experiences and feelings in life.	1. Review mindfulness and practice mindful hand thoughts 2. Recall the experience of failure due to avoidance through the “Trying strategies and their long-term effects” table 3. “Push the folder” exercise	Accept the useless emotions and feelings in hoarding behavior with an open and inclusive mind.
4. Cognitive defusion	Members reflect on what they think when they allow themselves to have hoarding behaviors and understand what the nature of the thoughts are	1. Metaphor of “Passengers on the Bus” 2. “Lemon” and “milk” exercises 3. “Computer Display Sense” exercise 4. “Leaves floating in the stream” meditation practice	Meditation practice to improve your ability to defusion.
5. Self-as-context	1. Distinguish between the conceptualized self, the observed self and the experiential self 2. Do not get trapped in the conceptualized self	1. “Slit lamp” metaphor 2. The metaphorical story of sky and weather 3. “The process of you” exercise	Combining cognitive defusion and self-as-context in one exercise.
6. Finding values	1. Clarify the difference between goals and values 2. Facilitate finding your own values	1. “compass, map and steering wheel” metaphor 2. “Two kids go to the zoo” metaphor 3. Give yourself an “80th birthday” 4. “Value Card Classification” exercise	Set some short-term, medium-term and long-term goals according to the SMART principle.
7. Commitment to action	1. Clarify that action is key to reducing hoarding behavior 2. Translate values into sustainable, developmental patterns of behavior 3. Develop your own plan	1. “Alpine skiing” metaphor 2. “climb the mountain” metaphor 3. “Intention and Action Plan” exercise 4. “Jumping” exercise 5. Practice from FEAR to DARE	Perform two value-based actions centered around reducing hoarding during the following week, and observe and record any thoughts, feelings or emotions that appear before, during and after you perform each value-based action.
8. Looking back at the way we came	1. Summarize the gains and feelings, discuss the changes brought by the group activities, and make hopes for the future 2. Handling the parting emotions of the members	1. Each person reviews his or her performance in the activity, summarizes and shares the harvest 2. Members send their blessings to each other	Achieve three value-oriented short-term goals around reducing hoarding behavior next month.

#### REBT condition

The intervention program was based on ABC theory, with reference to the book *How to make yourself happy and remarkably less disturbable* (75) and *Rational Emotive Behavior Therapy* (76). A total of 8 weeks of intervention programs were designed. See Table 2 for the specific intervention items.

**Table 2:** Rational emotive behavior therapy intervention program.

**Theme**	**Purpose of the activity**	**Main activities**	**Homework**
1. You and I get together	1. Explain the purpose, format and principles of group therapy, and familiarize members with each other 2. Understand Rational Emotive Behavior Therapy	1. Through activities such as “group hugging” and “snowballing”, members become familiar with each other 2. Clarify the purpose of group activities 3. Introduce rational emotional behavior therapy	Read about Rational Emotive Behavior Therapy
2. Knowing yourself	1. visually show how members see themselves in the moment 2. Experience the feeling of disconnection and hoarding	1.Warm-up activity 2. Draw the ideal room	Look back at the room you drew and find more reasons to justify it.
3. Analyze yourself	1. Help members discover the existence of beliefs and perceptions, and the impact they have 2. Understand the characteristics of irrational beliefs	1. Warm-up activity 2. Perform the “ABC triple” exercise without prompting 3. Share the results of the exercise, find irrational beliefs, and summarize the characteristics of irrational beliefs	Reinforce the “three-stage” exercise, and try to find your irrationality behind your bad emotions and behaviors.
4. Be happy with yourself	1. Make members aware that hoarding behavior comes from insecurity 2. Let members discover the importance of self-acceptance	1. Warm-up activity 2. Practice the idea of “self-acceptance” Conduct a self-acceptance exercise 3. Practice the idea of “self-acceptance	Conduct self-acceptance exercises.
5. Debate with irrationality	Let members learn how to debate their own irrational beliefs about hoarding in the process of exploration	1. Warm-up activity 2. Without prompting, members explore solutions on their own and share them with each other to verify if they work 3. Summarize ways to debate with irrational beliefs	Practice rebutting irrational beliefs related to self.
6. Reshape yourself	Integrate the concept of hoarding into the “ABCDE” theory to address hoarding behavior in a holistic and systematic way	1. Warm-up activity 2. Complete the “ABCDE” form 3. Reasonable Emotional Imagery Exercise	Continuous practice of rational emotional imagery techniques.
7. Positive Action	1. Make members acknowledge that problems are normal and not to be avoided 2. Help members to take positive action to reduce hoarding behavior even when they encounter difficulties	1. Warm-up activity 2. Action plan exercise	Practice the methods you learned in group counseling to reduce hoarding behavior.
8. Looking back at the way we came	1. Summarize the gains and feelings 2. Discuss the changes brought about by the group activities and make expectations for the future 3. Handling the parting emotions of the members	1. Each person reviews his or her performance in the activity summarizes and shares the gains 2. Send blessings to each other	Apply the skills learned from group counseling to future life.

### Measures

#### Primary outcome measure

##### Saving inventory-revised (SI-R)

This study used the Chinese SI-R to measure hoarding (77, 78). The scale consists of 21 items (e.g., “How distressing do you find the task of throwing things away”) and is scored on a 5-point scale from 0 (not at all) to 4 (very much so). The scale includes two dimensions: acquisition-difficulty discarding and clutter. Total sum scores and the score of each dimension were calculated. The Cronbach's α for this study was 0.95.

#### Secondary outcome measures

##### Obsessive-compulsive symptom scale (OCSS)

As hoarding has been considered a subtype of OCD (1), obsessive-compulsive symptoms were measured as a secondary outcome. The OCSS is a Chinese scale (79) with 18 items (e.g., “Washing hands frequently and more often and for a longer time than the average person.”) and is scored on a 5-point scale from 1 (not at all) to 5 (always). Total sum scores were used. The Cronbach's alpha coefficient for this study was 0.85.

##### Difficulties in emotion regulation scale (DERS)

The Chinese version of the DERS (80, 81) was used to measure individuals' difficulties in emotion regulation. The scale has 27 items (e.g., “When I'm upset, I feel guilty for feeling that way”) and is scored on a 4-point scale from 1 (not at all) to 4 (largely). Total sum scores were used. The Cronbach's alpha coefficient for this study was 0.86.

##### Experiences in close relationships inventory-attachment anxiety subscale (ECR)

The Chinese version of the ECR (82) was used to measure individuals' attachment anxiety for their relationship with their father, mother, best friend and romantic partner. The scale has 18 items (e.g., “I'm a little worried about losing my lover”) and is scored on a 7-point scale from 1 (strongly disagree) to 7 (strongly agree). Total sum scores were used. The Cronbach's alpha coefficient for this study was 0.94.

##### Depression anxiety stress scales (DASS-21)

The DASS-21 was designed to measure individual psychological distress, i.e., depression, anxiety, and stress (83). The revised Chinese version was applied in this study, with twenty-one items and three dimensions (84). A 4-point scale ranging from 0 (“did not apply to me”) to 3 (“applied to me very much”) is presented to each item, with higher scores indicating severe psychological distress. Total sum scores and the score of each dimension were calculated. Cronbach's alpha for the total scale and its subscales, i.e., depression, anxiety, and stress, were 0.84, 0.91, 0.85, and 0.82, respectively.

#### Process outcome measures

##### Acceptance and action questionnaire II (AAQ-II)

Psychological flexibility was assessed using the Chinese AAQ-II (85). This questionnaire included seven items (e.g., “I worry that I can't control my worries and feelings”) and was rated on a 7-point Likert scale ranging from 1 (never) to 7 (always). The scale has shown good reliability and validity in China (36). Higher AAQ scores indicate lower levels of psychological flexibility or higher levels of experienced avoidance. Total sum scores were used. In the present study, Cronbach's α was 0.89.

##### Cognitive fusion questionnaire (CFQ)

The CFQ is a 9-item scale that assesses cognitive confusion (86). The revised Chinese version with 9 items (e.g., “I tend to get very entangled in my thoughts”) was used in this study (87). The scale presents a 7-point response scale from 1 (never true) to 7 (always true). Higher scores indicate higher levels of cognitive confusion. Total sum scores were used. In this study, Cronbach's alpha was 0.96.

### Statistical analyses

Data entry and statistical analysis were performed using SPSS 22.0. The success of the randomization process was first tested by running a one-way ANOVA to test for differences in pre-measure across the three groups (ACT group, REBT group, and control group). Then, analysis of covariance and Bonferroni post hoc comparisons were applied to determine the differences in post-measure across the three groups. Effect size was reported as η^2^ (0.04 = small effect; 0.25 = medium effect; 0.64 = large effect (88).

Simple mediation models were performed with psychological flexibility or cognitive fusion as the mediator, group condition (ACT/REBT vs. control) as the independent variable, baseline values of the outcome and mediator as covariates, and post-intervention values of the primary or secondary variable as the dependent variable. Mediation was examined with a bootstrapping method and the PROCESS macro for SPSS (89). Parameter estimates were based on 5,000 resamples, and 95% bias-corrected confidence intervals were computed to determine the statistical significance of indirect effects.

## Results

One-way ANOVA results showed that there were no significant differences between the three groups on pre-measures regarding hoarding behaviors, psychological flexibility, cognitive fusion, OCD, negative affect, attachment anxiety, and difficulty in emotion regulation. However, each variable in the three groups differed significantly on the post-measures. For more details, they can be seen in Table 3.

**Table 3:** Descriptive statistics and one-way ANOVA.

**Variables**	**Phases**	**Descriptive Statistics (M** ±**SD)**			**Between groups**	
		**ACT**	**REBT**	**Control**	**F**	***P* value**
SI-R	Pre-test	46.16 ± 2.95	46.38 ± 3.18	46.06 ± 3.07	0.13	0.874
	Post-test	38.04 ± 4.13	41.55 ± 4.77	45.13 ± 5.76	23.66	< 0.001
A-DD	Pre-test	30.56 ± 4.09	29.57 ± 4.29	29.72 ± 3.93	0.77	0.464
	Post-test	26.13 ± 4.68	27.30 ± 5.18	29.40 ± 4.73	5.35	0.006
Clutter	Pre-test	15.59 ± 2.46	16.81 ± 3.23	16.34 ± 2.68	2.12	0.124
	Post-test	11.91 ± 1.81	14.26 ± 3.66	15.72 ± 3.69	16.62	< 0.001
AAQ	Pre-test	26.48 ± 4.05	25.36 ± 5.90	25.77 ± 6.03	0.50	0.609
	Post-test	22.07 ± 4.53	23.09 ± 5.96	23.50 ± 5.76	3.97	0.021
CFQ	Pre-test	36.61 ± 5.39	36.21 ± 6.70	35.04 ± 8.21	0.65	0.523
	Post-test	28.52 ± 5.15	32.04 ± 4.94	34.60 ± 7.29	12.26	< 0.001
DASS	Pre-test	48.20 ± 10.70	51.32 ± 11.05	47.45 ± 10.62	1.69	0.188
	Post-test	32.20 ± 11.48	42.47 ± 9.63	49.43 ± 10.29	31.35	< 0.001
Depression	Pre-test	15.51 ± 5.40	17.53 ± 6.44	15.49 ± 4.69	2.52	0.084
	Post-test	9.96 ± 4.98	14.77 ± 6.36	19.96 ± 6.57	31.69	< 0.001
Anxiety	Pre-test	16.00 ± 5.43	17.19 ± 6.26	18.09 ± 5.09	2.31	0.103
	Post-test	10.76 ± 6.80	13.28 ± 5.10	14.94 ± 5.39	6.08	0.003
Stress	Pre-test	16.91 ± 5.41	17.49 ± 5.70	15.49 ± 4.69	1.78	0.172
	Post-test	11.49 ± 4.99	14.43 ± 5.50	14.53 ± 4.95	5.13	0.007
OCSS	Pre-test	53.93 ± 8.19	54.64 ± 7.52	56.55 ± 7.67	1.41	0.249
	Post-test	48.15 ± 8.51	50.79 ± 7.86	54.87 ± 7.86	8.11	< 0.001
ECR	Pre-test	76.66 ± 18.79	74.79 ± 14.19	76.98 ± 18.92	0.22	0.806
	Post-test	66.36 ± 17.74	69.04 ± 12.89	77.47 ± 16.85	6.32	0.002
DERS	Pre-test	80.52 ± 8.30	83.79 ± 6.27	82.09 ± 6.38	2.49	0.087
	Post-test	74.20 ± 9.62	76.06 ± 6.55	81.19 ± 7.51	9.26	< 0.001

SI-R, the scores of Saving Inventory-revised; A-DD, (Acquisition - Difficulty Discarding) the subscale scores of SI-R; Clutter, the subscale scores of SI-R; AAQ, the scores of Acceptance and Action Questionnaire–II; CFQ, the scores of Cognitive Fusion Questionnaire; DASS, the scores of Depression Anxiety Stress Scale; Depression, the Depression subscale scores of DASS; Anxiety, the Anxiety subscale scores of DASS; Stress, the Stress subscale scores of DASS; OCSS, the scores of Obsessive-Compulsive Symptom Scale; ECR, the scores of Experiences in Close Relationships Inventory-Attachment Anxiety Subscale; DERS, the scores of Difficulties in Emotion Regulation Scale.

To accurately test and compare the differences in post-intervention between the three groups, posttest scores for each variable were used as the dependent variable, pretest scores were used as covariates, and the group was used as the independent variable for analysis of covariance and Bonferroni *post-hoc* comparisons. None of the interactions between the independent variables and the pretest scores of each outcome variable were significant, which satisfied the conditions of the analysis of covariance. The results indicated that hoarding behaviors, psychological flexibility, cognitive fusion, negative affect (depression, anxiety, and stress), OCD, attachment anxiety, and difficulties in emotion regulation differed significantly in all three groups, as shown in Table 4.

**Table 4:** The results of one-way ANCOVA.

**Dependent variable**	***SS_*W*_***	***SS_*B*_***	***df***	***MS***	**F**	***P*-value**	**η^2^**
SI-R	1981.34	1188.56	2	594.28	40.49	< 0.001	0.38
A-DD	1618.36	360.61	2	180.31	15.04	< 0.001	0.18
Clutter	1271.44	289.91	2	144.96	15.39	< 0.001	0.19
AAQ	399.42	355.18	2	177.59	60.02	< 0.001	0.47
CFQ	3806.06	1016.31	2	508.025	18.02	< 0.001	0.21
DASS	7033.37	7272.27	2	3636.13	69.79	< 0.001	0.51
Depression	1706.87	1390.87	2	695.44	55.01	< 0.001	0.45
Anxiety	3075.99	565.56	2	282.63	12.40	< 0.001	0.16
Stress	1350.46	394.82	2	197.41	19.73	< 0.001	0.23
OCSS	1664.61	412.10	2	206.05	16.71	< 0.001	0.20
ECR	5746.91	2959.72	2	1479.86	34.76	< 0.001	0.34
DERS	1307.90	1228.75	2	614.37	63.42	< 0.001	0.48

SI-R, the scores of Saving Inventory-revised; A-DD, (Acquisition—Difficulty Discarding) the subscale scores of SI-R; Clutter, the subscale scores of SI-R; AAQ, the scores of Acceptance and Action Questionnaire–II; CFQ, the scores of Cognitive Fusion Questionnaire; DASS, the scores of Depression Anxiety Stress Scale; Depression, the Depression subscale scores of DASS; Anxiety , the Anxiety subscale scores of DASS; Stress, the Stress subscale scores of DASS; OCSS, the scores of Obsessive-Compulsive Symptom Scale; ECR, the scores of Experiences in Close Relationships Inventory-Attachment Anxiety Subscale; DERS, the scores of Difficulties in Emotion Regulation Scale; SS_B_, Sum of Squares Between groups; SS_W_, Sum of Squares Within groups; MS, Mean Square; η^2^, *SS*_*B*_/(*SS*_*B*_ + *SS*_*W*_).

**Figure 2:**
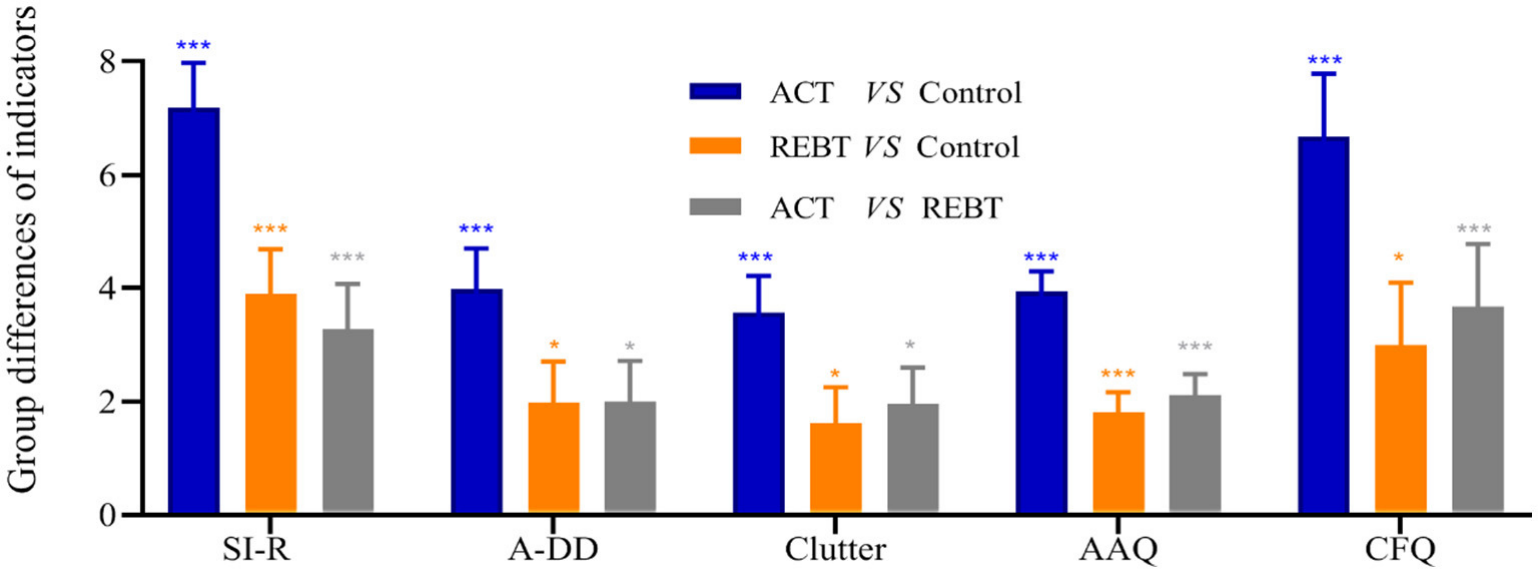
The results of Bonferroni's *post hoc* comparison (SI-R, A-DD, Clutter, AAQ, and CFQ). ^*^p < 0.05; ^***^p < 0.001; Blue bar, Difference between ACT group and Control group; Orange bar, Difference between REBT group and Control group; Gray bar, Difference between ACT group and REBT group. SI-R, the scores of Saving Inventory-revised; A-DD, (Acquisition-Difficulty Discarding) the subscale scores of SI-R; Clutter, the subscale scores of SI-R; AAQ, the scores of Acceptance and Action Questionnaire–II; CFQ, the scores of Cognitive Fusion Questionnaire.

**Figure 3:**
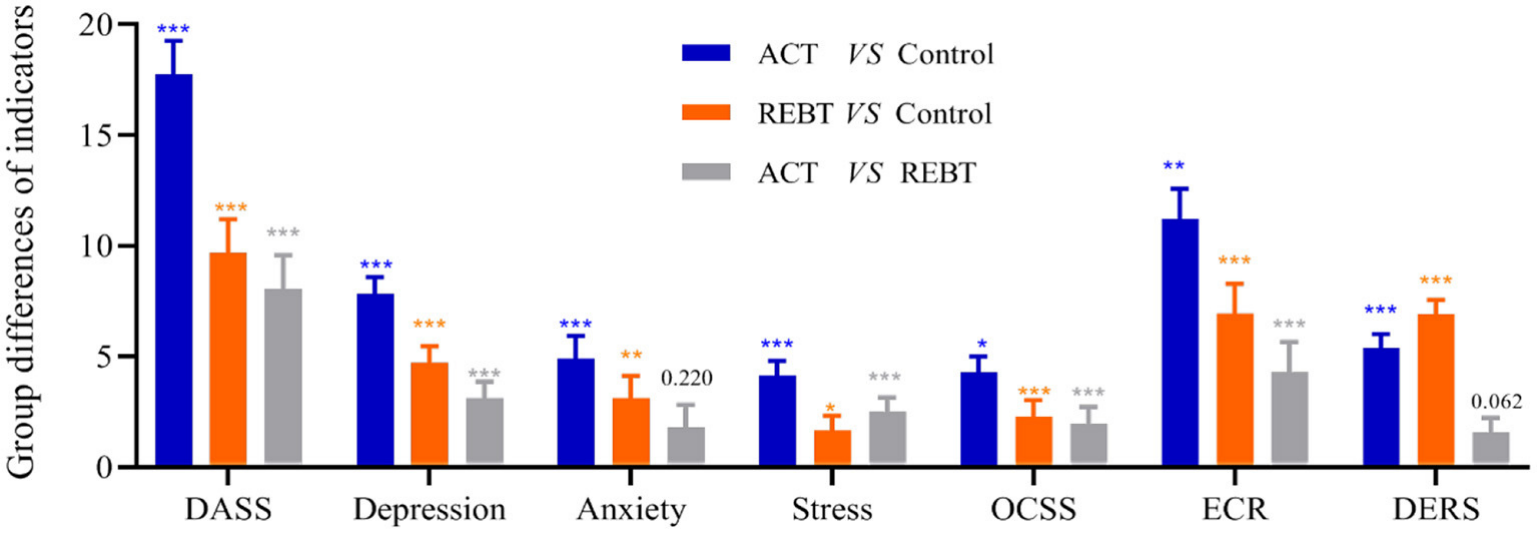
The results of Bonferroni's *post hoc* comparison (DASS, Depression, Anxiety, Stress, OCSS, ECR, and DERS). ^*^p < 0.05; ^**^p < 0.01; ^***^p < 0.001; DASS, the scores of Depression Anxiety Stress Scale; Depression, the Depression subscale scores of DASS; Anxiety, the Anxiety subscale scores of DASS; Stress, the Stress subscale scores of DASS; OCSS, the scores of Obsessive-Compulsive Symptom Scale; ECR, the scores of Experiences in Close Relationships Inventory-Attachment Anxiety Subscale; DERS, the scores of Difficulties in Emotion Regulation Scale.

Figures 2, 3 show the results of the Bonferroni *post-hoc* test for each outcome variable in the three groups. This result shows that after controlling for baseline levels of each variable, participants who received the ACT intervention and the REBT intervention showed significantly greater improvements in each outcome than those in the control group. In addition, the ACT group was significantly better than the REBT group in improving psychological flexibility and reducing hoarding (acquisition-difficulty discarding, clutter), cognitive fusion, negative affect (depression and stress), and OCD. However, for Anxiety (ACT vs. REBT, *p* = 0.220) and ECR-AA (ACT vs. REBT, *p* = 0.062), there was no significant difference in the effects of the two interventions.

The results of the mediation analyses showed that the treatment condition indirectly influenced hoarding behaviors (SI-R), negative affect (DASS), and attachment anxiety (ECR-AA) through its effect on psychological flexibility (AAQ-II) but not through the cognitive fusion change process (CFQ). As seen in Table 5, however, psychological flexibility does not mediate the effect of treatment conditions on OCD and difficulties in emotion regulation. The indirect effect of treatment condition on hoarding behaviors, negative affect, and attachment anxiety through AAQ-II was, respectively estimated as −1.48 (SE = 0.63, 95% CI: −2.84 −0.37), −3.49 (SE = 1.16, 95% CI: −6.14, −1.62),−2.16 (SE = 0.82, 95% CI: −3.98, −0.83), indicating that hoarding behaviors, negative affect, and attachment anxiety among participants in ACT/REBT were, on average, 1.48, 3.49, and 2.16 units lower than that of participants in the control group as a result of the indirect effect through psychological flexibility. The bias-corrected bootstrap confidence intervals for the indirect effects of psychological flexibility based on 5,000 bootstrap samples did not contain zero, suggesting that these effects were significant.

**Table 5:** The specific indirect effects of the intervention on each outcome measure through changes in psychological flexibility.

			**Bca 95%**	
	**Point estimate**	**Boot SE**	**Lower CI**	**Upper CI**
**Model 1. Hoarding behaviors**				
Indirect effect	−1.48	0.63	−2.84	−0.37
Total effect	−5.49	0.72	−6.93	−4.06
**Model 2**. N**egative affect (DASS)**				
Indirect effect	−3.49	1.16	−6.14	−1.62
Total effect	−13.74	1.43	−16.57	−10.91
**Model 3. Attachment anxiety**				
Indirect effect	−2.16	0.82	−3.98	−0.83
Total effect	−9.06	1.21	−11.45	−6.68
**Model 4**. O**bsessive-compulsive symptom**				
Indirect effect	−0.62	0.47	−1.71	0.14
Total effect	−3.22	0.65	−4.5	−1.93
**Model 5. Difficulties in emotion regulation**				
Indirect effect	0.19	0.47	−0.76	1.11
Total effect	−6.15	0.57	−7.28	−5.03

Boot SE, Bootstrap standard error; Bca, bias corrected and accelerated; CI, confidence interval.

## Discussion

In this study, ACT and REBT were found to reduce hoarding compared to the control group. Specifically, ACT and REBT improved individuals' psychological flexibility and reduced cognitive fusion, acquisition-difficulty discarding, clutter, negative affect (anxiety, depression, stress), attachment anxiety, obsessive-compulsive disorder, and difficulty in emotion regulation. In addition, ACT was more effective than REBT in improving psychological flexibility and reducing hoarding, cognitive fusion, depression, stress, and obsessive-compulsive disorder; there were no significant differences between the two in anxiety and emotion regulation difficulties. Finally, psychological flexibility is a mediator of the effect of ACT and REBT on some behavioral and psychological outcomes (hoarding behaviors, negative affect, attachment anxiety).

ACT has been found to alleviate individuals' OCD (90, 91), negative affect (92–94), hoarding (66, 67), difficulties with emotion regulation (95, 96), and attachment anxiety (97), which are consistent with the findings of the present study. According to the cognitive model of hoarding, the performance of hoarding stems from various avoidance behaviors (3, 30). Patterns of behavioral avoidance have been observed in most hoarding patients (25, 98). Additionally, the primary goal of ACT is to reduce cognitive fusion and experiential avoidance and increase psychological flexibility so that individuals can reduce their avoidance behaviors and act in accordance with their values (32, 36, 58).

Previous studies have suggested that other therapies can also affect psychological flexibility, including other forms of CBT, such as exposure and response/or ritual prevention (99, 100), which is also consistent with our study. The present study found that REBT, as a form of CBT, can increase psychological flexibility and reduce cognitive fusion. Theoretical studies of hoarding have found that irrational perceptions of possessions are an important cause of hoarding (3). REBT focuses on a person's irrational beliefs, which is consistent with the mechanisms that produce hoarding. The most distinctive feature of REBT is that it helps individuals change their rigid and extreme beliefs to flexible and non-extreme beliefs (101) with various cognitive skills (49, 52). However, cognitive skills need to be practiced continuously (102, 103). People may be negligent in performing cognitive practice, making it less useful than ACT for improving psychological flexibility. This may explain why ACT is more effective in improving psychological flexibility and reducing hoarding, cognitive fusion, depression, stress, and OCD.

Additionally, some studies focusing on mechanisms of change have suggested that the effect of ACT or CBT on outcome variables is mediated by its impact on psychological flexibility (68–70, 104). The present study also found that psychological flexibility played a mediating role in the efficacy of ACT/REBT on the outcome variables (i.e., hoarding behaviors, negative affect, and attachment anxiety). Psychological flexibility, as a fundamental aspect of health, is a cornerstone of healthy personal and social functioning and one of the foremost goals of human existence (105). Empirical research has shown that psychological flexibility is implicated in a variety of psychopathological and functional outcomes (106). Models of developmental psychopathology (107) and theories of mechanisms of change in psychotherapy (108) conclude that psychological flexibility in emotion, behavior, and cognition predicts successful psychosocial adjustment and treatment (106). Therefore, enhancing psychological flexibility is a primary mechanism by which evidence-based psychotherapies produce adaptive behavioral change and related outcomes (39, 109, 110).

The present study did not find a mediating role of cognitive fusion. Future research should explore the possible mediating roles of other process variables in the effects of various forms of CBT on psychological and behavioral outcomes related to hoarding. Such process-based research may be better able to distill potent components from ACT and other formats of CBT to develop a coherent intervention framework for hoarding in the service of streamlining treatment development, dissemination, and implementation (66, 111).

There are some limitations in this study. The present study did not carry out follow-up measures to examine the durability of the effects of the intervention study, and there is a lack of follow-up studies on the stability of the intervention effect. Second, this study measured psychological flexibility using the Acceptance and Action Questionnaire II (AAQ-II), which measures only one dimension of psychological flexibility (112, 113). In addition, all studies were based on self-reported data. Given these limitations, the findings from this study should be interpreted with caution. Because of the theoretical importance of hoarding behaviors in current psychological models, further research is needed to reveal neural mechanisms associated with the cognitive representation of hoarding behaviors pre- and post-interventions. Future research is needed to explore the effects of other forms of CBT-based group interventions (e.g., mindfulness-based stress reduction, mindfulness-based cognitive therapy or dialectical behavioral therapy) on hoarding behaviors and the neural mechanisms involved.

Overall, current findings provide preliminary support for the efficacy of ACT and REBT for hoarding and associated impairment in China. We found that ACT was more effective than REBT in improving psychological flexibility and reducing hoarding, cognitive fusion, depression, stress, and obsessive-compulsive disorder. Furthermore, this study examined the mediating role of psychological flexibility as a process variable. A test of mediating effects would be helpful in understanding the mechanisms of CBT in non-Western countries and the role of psychological flexibility in individual psychological and behavioral changes.

## Data availability statement

The datasets used and/or analyzed during the current study are available from the corresponding author upon reasonable request.

## Ethics statement

The studies involving human participants were reviewed and approved by the Ethical Committee of Anhui Normal University. The participants provided their written informed consent to participate in this study.

## Author contributions

SF: conceptualization, methodology, supervision, design experiments, validation, data collection, data curation, formal analysis, visualization, writing—original draft, writing—review and editing, and project administration. DD: design experiments, formal analysis, writing—original draft, and writing—review and editing. RZ: design experiments, implementation of experiments, data collection, data curation, formal analysis, and writing—original draft. MH: design experiments, implementation of experiments, data collection, data curation, formal analysis, visualization, and writing—original draft. All authors contributed to the article and approved the submitted version.
